# Evaluation of an 8-Week Vegan Diet on Plasma Trimethylamine-N-Oxide and Postchallenge Glucose in Adults with Dysglycemia or Obesity

**DOI:** 10.1093/jn/nxab046

**Published:** 2021-03-30

**Authors:** Stavroula Argyridou, Melanie J Davies, Gregory J H Biddle, Dennis Bernieh, Toru Suzuki, Nathan P Dawkins, Alex V Rowlands, Kamlesh Khunti, Alice C Smith, Thomas Yates

**Affiliations:** Diabetes Research Centre, University of Leicester, Leicester General Hospital, Leicester, United Kingdom; National Institute for Health Research Leicester Biomedical Research Centre, University of Leicester, Leicester, United Kingdom; Diabetes Research Centre, University of Leicester, Leicester General Hospital, Leicester, United Kingdom; National Institute for Health Research Leicester Biomedical Research Centre, University of Leicester, Leicester, United Kingdom; Diabetes Research Centre, University of Leicester, Leicester General Hospital, Leicester, United Kingdom; National Institute for Health Research Leicester Biomedical Research Centre, University of Leicester, Leicester, United Kingdom; Department of Cardiovascular Sciences, University of Leicester, National Institute for Health Research Leicester Biomedical Research Centre, Leicester, United Kingdom; Department of Cardiovascular Sciences, University of Leicester, National Institute for Health Research Leicester Biomedical Research Centre, Leicester, United Kingdom; Diabetes Research Centre, University of Leicester, Leicester General Hospital, Leicester, United Kingdom; National Institute for Health Research Leicester Biomedical Research Centre, University of Leicester, Leicester, United Kingdom; Diabetes Research Centre, University of Leicester, Leicester General Hospital, Leicester, United Kingdom; National Institute for Health Research Leicester Biomedical Research Centre, University of Leicester, Leicester, United Kingdom; Diabetes Research Centre, University of Leicester, Leicester General Hospital, Leicester, United Kingdom; National Institute for Health Research Collaboration for Leadership in Applied Health Research and Care–East Midlands, University of Leicester, Leicester, United Kingdom; Leicester Kidney Lifestyle Team, Department of Health Sciences, University of Leicester, Leicester, United Kingdom; Diabetes Research Centre, University of Leicester, Leicester General Hospital, Leicester, United Kingdom; National Institute for Health Research Leicester Biomedical Research Centre, University of Leicester, Leicester, United Kingdom

**Keywords:** metabolites, trimethylamine-N-oxide, type 2 diabetes, vegan diet

## Abstract

**Background:**

Trimethylamine N-oxide (TMAO), a metabolite generated by the gut in response (in part) to meat consumption, is linked to poor cardiometabolic health.

**Objectives:**

We investigate the effect of an 8-week vegan diet, followed by a 4-week period of unrestricted diet, on glucose tolerance and plasma TMAO in human omnivores with obesity or dysglycemia.

**Methods:**

This interventional single-group prospective trial involved 23 regular meat eaters with dysglycemia [glycated hemoglobin ≥ 5.7% and ≤8% (39–64 mmol/mol)], or obesity (ΒΜΙ ≥ 30 kg/m^2^) aged 57.8 ± 10.0 years. Participants [14 men (60.9%) and 9 women (39.1%)] were supported in following a vegan diet for 8 weeks, followed by 4 weeks of unrestricted diet. The primary outcomes (plasma TMAO and glucose) were assessed at baseline, during the vegan diet (weeks 1 and 8), and after the unrestricted diet period (week 12). TMAO was assessed after fasting and glucose was measured as a time-averaged total AUC using a 180-minute oral-glucose-tolerance test. Generalized estimating equation models with an exchangeable correlation structure were used to assess changes from baseline, adjusting for age, sex, ethnicity, and weight.

**Results:**

TMAO levels (marginal mean) were reduced after weeks 1 and 8 of a vegan diet compared to baseline, from 10.7 (97.5% CI, 6.61–17.3) μmol/L to 5.66 (97.5% CI, 4.56–7.02) μmol/L and 6.38 (97.5% CI, 5.25–7.74) μmol/L, respectively; however, levels rebounded at week 12 after resumption of an unrestricted diet (17.5 μmol/L; 97.5% CI, 7.98–38.4). Postprandial glucose levels (marginal means) were reduced after weeks 1 and 8 compared to baseline, from 8.07 (97.5% CI, 7.24–8.90) mmol/L to 7.14 (97.5% CI, 6.30–7.98) mmol/L and 7.34 (97.5% CI, 6.63–8.04) mmol/L, respectively. Results for glucose and TMAO were independent of weight loss. Improvements in the lipid profile and markers of renal function were observed at week 8.

**Conclusions:**

These findings suggest that a vegan diet is an effective strategy for improving glucose tolerance and reducing plasma TMAO in individuals with dysglycemia or obesity. This study was registered at clinicaltrials.gov as NCT03315988.

## Introduction

The number of people with diabetes worldwide quadrupled between 1980 and 2014, from 108 to 422 million, 85–90% of whom have type 2 diabetes mellitus (1), which accounts for around 10% of global health-care expenditures (2). A large portion of this burden is due to managing the elevated prevalence of cardiovascular disease (CVD), which is a major cause of mortality in individuals with type 2 diabetes (3). Type 2 diabetes prevention and management strategies are routinely focused on weight management, improving glycemic control, and managing CVD risk factors (1).

Current dietary guidance for both management and prevention of type 2 diabetes is based on reducing energy and saturated fat intake, and increasing dietary fiber (4). Observational evidence has also suggested that plant-based diets are additionally associated with improved lipid profiles, lower prevalences of heart disease, and a reduced risk of cancer, with intervention studies further suggesting that vegan diets may be more efficacious in the management of type 2 diabetes than diets based on current dietary guidance (5–7). Coupled with the WHO classification of processed meat as a class 1 carcinogen and red meat as a class 2A carcinogen (8), this has led to growing public interest in the health benefits of plant-based diets. However, there is a lack of consensus across health-care organizations. which in part may be attributed to the lack of intervention studies or elucidated mechanisms linking high levels of meat consumption to poor cardiometabolic health. Higher production of trimethylamine N-oxide (TMAO) with high meat consumption could underlie poor cardiovascular health (9). TMAO is synthesized in the liver from trimethylamine (TMA) through the action of flavin-containing monooxygenase 3 (9). TMA is generated by gut microbiota in response to nutrients for which meat and eggs are the major dietary sources, such as choline and carnitine (9). Interest in and understanding of the role of TMAO in health and disease has increased over the last decade, with higher TMAO levels consistently associated with an elevated risk of CVD and mortality (10, 11). In addition, those with dysglycemia have elevated levels of TMAO, while animal studies have suggested that ingestion of TMAO may promote insulin resistance and impaired glucose tolerance (12).

Despite the mounting evidence linking TMAO to adverse health outcomes, much less is known about the impact of dietary interventions on circulating TMAO levels. Intervention studies to date have predominately focused on elevations in TMAO after ingesting animal products or choline/carnitine supplements in healthy populations (9, 13–15). There has been a lack of research investigating whether eliminating animal products from the diet can reduce TMAO levels along with improving cardiovascular health. Whether eliminating the primary dietary factors known to contribute to the production of TMAO actively leads to a meaningful reduction in circulating TMAO, and the impact of such an intervention in those at risk of cardiometabolic disease, is not known.

The aim of this study was to test the hypothesis that eliminating animal products from the diet will result in reduced levels of TMAO and improved glucose tolerance in individuals with obesity or dyglycemia.

## Methods

### Design

This was a single-center, interventional, single-group, prospective efficacy trial conducted at the Leicester Diabetes Centre, Leicester, UK. The study consisted of 3 phases: baseline, an 8-week vegan diet, and 4 weeks of unrestricted diet resumption. Measurement visits were conducted at baseline (visit 1; week 0), at 1 week after introducing the vegan diet (visit 2; week 1), at completion of the vegan diet (visit 3; week 8), and after 4 weeks of unrestricted diet resumption (visit 4; week 12). Recruitment and study procedures were conducted between October 2017 and January 2019. This study was approved by the West of Scotland Research Ethics Service (17/WS/0184). All participants gave written informed consent.

### Participants

Recruitment was conducted through primary care, where patient databases were searched for those meeting the following inclusion criteria: aged between 18 and 75 years; either overweight (white participants, BMI ≥ 25 kg/m^2^ to < 30 kg/m^2^; South Asian/black participants, ≥23 kg/m^2^ to < 27.5 kg/m^2^) with glycated hemoglobin (HbA1c) ≥5.7% and ≤8% (39–64 mmol/mol) identified within the last 36 months or obese (white participants, BMI ≥ 30 kg/m^2^; South Asian/black participants, ≥ 27.5 kg/m^2^); not taking any form of glucose-lowering medication currently or within the last 60 days; no current or recent (within 6 months) oral antibiotic or steroid use; nonsmoker; and no ongoing CVD. In addition, participants self-reporting as vegan, vegetarian, or occasional meat eaters (<3 portions of meat/fish per week) were excluded.

### Intervention

After eligibility was confirmed and baseline assessments were completed, each participant had a 90-minute consultation with a research dietitian during which weekly menu plans and shopping lists were developed and personalized to the participants’ diet, personal preferences, and habitual energy intake. Information about the range of vegan products commonly available from popular supermarkets was provided, along with education on how to read and interpret food labels for the inclusion of animal products. Participants visited the center on 2 separate occasions during the intervention (week 1 and week 8 of the vegan diet). In addition, the research dietician provided individualized follow-on telephone support between face-to-face visits on a 10–14-day basis. After the end of the 8-week vegan diet, participants were asked to resume their normal, unrestricted diet. Participants were asked to continue their preexisting medication regimens throughout.

Portion sizes, energy intake, and the macronutrient content of the diet were unrestricted. A research dietician made unannounced telephone calls to each participant to administer a 24-hour diet recall. These recalls were not statistically analyzed, but allowed the investigators to check compliance rates and provide additional dietary counseling as needed. In addition, a 3-day dietary record was completed by each participant at baseline and at weeks 1, 8, and 12, with each record including 2 weekdays and 1 weekend day. For statistical purposes, nonadherence to the study protocol was defined according to the following criteria: *1*) 2 or more portions of meat, poultry, fish, or egg reported during 24-hour recalls; or *2*) if one-third or more of captured compliance scales (from 0, “non-compliant,” to 10, “fully compliant”) list a score of 5 or less.

### Primary Outcomes

We defined 2 primary outcomes a priori: glucose tolerance in an oral-glucose-tolerance test (OGTT) and fasting plasma TMAO.

#### Oral-glucose-tolerance test

The participants were requested to consume a vegan meal the evening before their baseline visit and then consume the same meal before subsequent visits to standardize the OGTT test. All participants were required to fast ≥10 hours prior to the OGTT (drinking water was allowed). Participants were asked to avoid general exercise and drinking alcohol, coffee, and caffeinated tea in the 48 hours before each OGTT. The OGTT involved collecting fasted blood samples and then ingesting 75 g of anhydrous glucose (300 ml Rapilose) with further blood samples collected at 30, 60, 120, and 180 minutes (16). Glucose was analyzed on the day of collection by the University Hospitals of Leicester pathology department using the Hexokinase method via an automated analyzer (ADVIA XPT clinical chemistry analyzer, Siemens Healthineers). Plasma was collected by centrifugation at 1500 x g for 10 minutes and stored in −80°C freezers. Insulin was analyzed from these plasma samples collectively at the end of the trial using Magnetic Luminex Assays (R&D Systems). Each sample was run in duplicate to ensure reliability of readings, and duplicate sample values with ≥20% variability were reanalyzed. OGTT data were used to derive the total AUC (tAUC) using the trapezoidal rule; tAUC values for insulin and glucose were then time averaged, as has been reported previously (17). Resulting values represent the mean glucose and insulin concentrations during the OGTT; values are therefore referred to as the average postprandial response.

#### TMAO measurement

Fasted blood samples collected during the OGTT were also used for the assessment of plasma TMAO levels. A sample analysis was conducted in duplicate using stable isotope dilution [D9-TMAO (98.0% purity)], followed by an ultra‐performance LC–tandem MS analysis. This was performed on a Shimadzu Nexera X2 LC-30AD coupled with a Shimadzu 8050 triple quadrupole mass spectrometer (Shimadzu Corp., Kyoto, Japan), using optimized conditions of a previously described method, which produced a CV of 3.6% (18).

### Secondary outcomes

#### Bio-anthropometric measurements

Information on current smoking status, family history of type 2 diabetes, medication status, and ethnicity was obtained after an interview-administered questionnaire with a health-care professional. Systolic and diastolic blood pressure measurements (mm Hg) were taken after participants had rested in a seated position for 5 minutes, using a digital monitor and a standard cuff maintained at the level of the heart. Three measurements were taken at 2-minutes intervals; the first measurement was disregarded, and the mean of the last 2 was used for analysis.

Body weight (Tanita TBE 611; Tanita) and height (Leicester Height Scale) were measured to the nearest 0.1 kg and 0.5 cm, respectively. The subsequent values were used to compute BMI (kg/m^2^). Waist circumference was measured over light clothing between the lower rib margin and the iliac crest and recorded to the nearest 0.5 cm.

Body composition was assessed using DXA (GE Lunar iDXA Series model) by a fully qualified technician. Each participant's body composition was measured at 2 visits (baseline and week 8). A DXA scan was used to assess each participant's body fat mass and fat-free mass, as well as body composition within segments.

Serum total cholesterol (esterase and cholesterol oxidase conversion, followed by a Trinder end point), HDL cholesterol (Trinder reaction following elimination of chylomicrons), triglycerides (GPO-Trinder), urea (urease enzymatic method), and creatinine (Jaffe reaction) were measured by individuals blinded to the participants within routine clinical laboratories at the University Hospitals of Leicester laboratories via an automated analyzer (ADVIA XPT clinical chemistry analyzer, Siemens Healthineers). HbA1c was analyzed using the HPLC method on a Tosoh G11 analyzer (Tosoh Corp., Shiba). Centrifuged plasma samples stored in −80°C freezers were analyzed for C-reactive protein using Magnetic Luminex Assays (R&D Systems). LDL cholesterol was calculated as: total cholesterol − (triglyceride/2.2) – HDL cholesterol. The estimated glomerular filtration rate (eGFR) was calculated by the modification of diet in a renal disease study equation: 186 × (creatinine/88.4) − 1.154 × (age) − 0.203 × (0.742 if female) × (1.210 if black) (19). The urea to creatinine ratio was estimated [urea/(creatinine/1000)].

#### Physical activity

The participants were asked to maintain their normal physical activity levels throughout the intervention. Physical activity was measured using an accelerometer worn 24 hours a day on the wrist (GENEActiv, Activinsights Ltd; ±8-g sensor). Raw acceleration data were captured at 20 Hz. Data were captured at baseline (2 weeks + 5 days) and throughout the intervention (8 weeks) and follow-up period (4 weeks). Data were processed in the GGIR open access package in R (RStudio, version 1.10–4; http:/cran.r-project.org) using methods previously described (20) to generate time spent inactive, in light intensity physical activity, and engaged in 1-minute bouts of moderate-to-vigorous physical activity.

### Statistical analysis

#### Sample size

A 20% reduction in glucose tAUC was anticipated as a conservative estimate for the intervention effect based on previous dietary interventions (5, 21). Therefore, given that the SD for glucose tAUC values has previously been found to represent 27% of the mean in those at high risk of type 2 diabetes (22), resulting in a standardized difference of 0.74, and assuming a significance of 0.025 (allowing for 2 primary outcomes), a power of 80%, and an intra-individual correlation of 0.5 across repeated measures, a total of 20 participants were required.

Using data generated from a previous study showing a reduction in TMAO of 1.74 μmol/L was associated with improved cardio-metabolic health with an SD of 2.06 μmol/L (23), this sample size also provides over 80% power to detect a meaningful change in TMAO, assuming a significance of 0.025 (allowing for 2 primary outcomes) and intra-individual correlation of 0.5 across repeated measures.

#### Analysis

This analysis was conducted using IBM SPSS Statistics (version 24.0). In order to investigate the effect of an 8-week vegan diet on postprandial glucose and TMAO levels compared to baseline, generalized estimating equation (GEE) models were used. GEE models were built with an exchangeable correlation structure to allow for analyses of repeated measurements at baseline, 1 week, 8 weeks, and 12 weeks. Postprandial glucose was analyzed using a normal distribution with an identity link, whereas TMAO displayed a skewed distribution and was therefore analyzed assuming a gamma distribution with an identity link. In order to investigate the extent to which changes in the primary outcomes are influenced by changes to body weight or lifestyle factors, 2 models were developed: Model 1 remained unadjusted for covariates, while Model 2 was adjusted for age, sex, ethnicity, and weight as repeated (time-varying) covariates. Significant main effects for the models were explored through post hoc tests comparing each follow-up to baseline. Secondary outcomes were analyzed using the same approach. A Pearson correlation matrix was used to investigate the correlation between the change in TMAO and change in postprandial glucose at each time point. We considered 2-tailed *P* values of 0.025 or less statistically significant for main effects to allow for the inclusion of 2 primary outcomes. Results are presented as means (97.5% CIs), unless stated otherwise.

#### Sensitivity analysis

In order to assess whether changes in glucose were independent of changes in TMAO and vice versa, models were mutually adjusted. Model 2 was also further adjusted for diet macronutrient composition or physical activity to assess whether changes in the primary outcomes were independent of any changes to macronutrient composition and physical activity.

## Results

**FIGURE 1:**
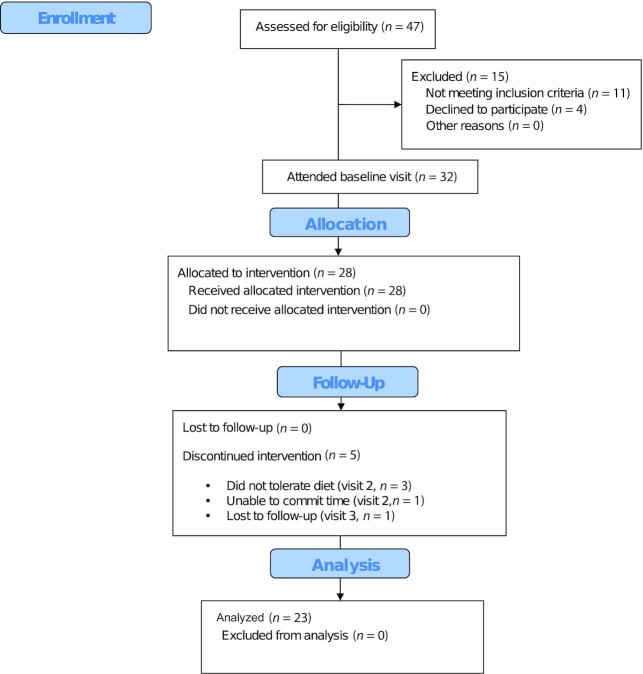
Flowchart of participants through the Plant Your Health trial. Numbers are original to this manuscript.

Invitation letters were sent to identified eligible participants (*n* = 918), and 47 replies were received. Of 47 individuals screened by telephone using a prescreening questionnaire, 32 met the inclusion criteria to attend the baseline visit. A total of 28 individuals were identified as eligible at the baseline visit (**Figure 1**). Reasons for exclusion were HbA1c values outside the required range (*n* = 1), a BMI lower than the required range (*n* = 1), and reluctance to change diet (*n* = 2). A total of 23 individuals completed the study. The other 5 participants withdrew from the study: 3 because they did not tolerate the diet, 1 due to being unable to commit time, and 1 due to loss to follow-up.

A total of 14 male and 9 female individuals aged 57.8 ± 10.0 years completed the trial. Among completers, medications remained unchanged throughout the intervention. The bio-anthropometric, physical activity, and dietary characteristics of included participants at baseline are displayed in **Table 1**. Participants had a median daily energy intake of 1799 kcal, with 50%, 34%, and 16% of energy intake coming from carbohydrates, fat, and protein, respectively. Over the 3-day food diary data collection period, participants also reported a median of 1 serving of meat per day, 1 serving of eggs every 3 days, and no servings of fish.

**TABLE 1:** Bio-anthropometric, physical activity, and dietary characteristics of the Plant Your Health study participants (*n* = 23)^1^

Characteristics	Baseline data
Age, y	57.8 ± 10.0
Sex, *n* (%)	
Men	14 (60.9)
Women	9 (39.1)
Smoking status, *n* (%)	
Never smoked	17 (73.9)
Ex-smokers	6 (26.1)
Ethnicity	
White European	13 (56.5)
Other	10 (43.5)
Medication, *n* (%)	
Statin	9 (39.1)
Antiplatelet	2 (8.7)
Anticoagulant	1 (4.3)
Diuretic	2 (8.7)
Alpha blocker	1 (4.3)
Beta blocker	1 (4.3)
Calcium channel blocker	8 (34.8)
Ace inhibitors	6 (26.1)
Anti-depressive drugs	6 (26.1)
Thyroid drugs	3 (13.0)
Daily nutritional supplements (Vitamin D)	4 (17.4)
Gastrointestinal medication	7 (30.4)
BMI, kg/m2	35.2 ± 6.6
Waist circumference, cm	112.1 ± 13.7
DXA^2^	
Fat mass, Kg	42.3 ± 15.5
Lean mass, Kg	50.1 ± 9.0
Visceral fat, Kg	2.1 ± 1.3
Total tissue fat, % body weight	45.0 ± 6.8
Systolic BP, mmHg	135 ± 16
Diastolic BP, mmHg	84 ± 13
HbA1c, %	6.0 ± 0.3
Serum biochemistry	
Total cholesterol, mmol/L	4.8 (4.2–6.0)
HDL cholesterol, mmol/L	1.3 (1.1–1.5)
LDL cholesterol, mmol/L	2.6 (2.2–3.7)
Triglycerides, mmol/L	1.6 (1.1–2.5)
Creatinine, μmol/L	70.0 (57.0–79.0)
Urea, mmol/L	5.1 (4.3–6.0)
UCR	56.5 (51.6–67.05)
eGFR, mL⋅min^−^1⋅1.73 m^−2^	96.7 (86.4–113.3)
Plasma biochemistry	
TMAO, μmol/L	7.7 (4.9–9.9)
CRP, mg/L	3.9 (1.6–5.5)
Average postprandial glucose response, mmol/l	7.9 (7.2–9.0)
Average postprandial insulin response, mIU/L	100.2 (64.8–135.5)
Dietary intake, unit/d	
Energy, Kcal/d	1799 (1410–2136)
Carbohydrate, g/d	214 (142–246)
Fiber, g/d	20 (15–27)
Non-starch polysaccharides, g/d	15 (11–20)
Protein, g/d	74 (62–98)
Fat, g/d	68 (52–83)
Saturated fat, g/d	22 (15–27)
Accelerometer variables^2^	
Wear time, h/day	24 (23–24)
Light‐intensity physical activity, min/day	165 (135–196)
MVPA, min/day	21 (13-38)
Inactive time, min/day	724 (665–777)

1 Values are means ± SDs or medians (IQRs), unless otherwise indicated. *n* = 23. BP, blood pressure; CRP, C-reactive protein; eGFR, estimated glomerular filtration rate; HbA1c, glycated hemoglobin; MVPA, moderate to vigorous physical activity; TMAO, trimethylamine-N-oxide; UCR, urea to creatinine ratio.

2 Inactive time was defined as up to 40 mg; light intensity as 40–100 mg; and MVPA as 100+ mg. MVPA included the total MVPA of at least 1-minute bouts.

### Dietary composition and compliance with the intervention

Dietary variables throughout the intervention are presented in **Table 2**. The mean compliance rating—evaluated using a compliance scale weekly throughout the intervention (a maximum of 10 points)—was 9.83 ± 0.56 (see **Supplemental Table 1**), with no participants meeting the nonadherence criteria of 2 or more portions of meat, poultry, fish, or egg during the previous 24 hours. In addition, no servings of meat were recorded during the food diary data collection in week 1 (visit 2) or week 8 (visit 3). The removal of meat from the diet resulted in a significant reduction to protein and saturated fat intakes during weeks 1 and 8, with fiber intake increased during week 1 (Table 2). Energy intake was significantly reduced during week 1, but not at other time points.

**TABLE 2:** The effect of an 8-week vegan diet on primary and secondary outcomes^1^

Variables	Model	*n*	Baseline	*n*	Week 1	*n*	Week 8	*n*	Week 12	*P* for model
Primary outcomes										
TMAO, μmol/L	1	23	10.7 (6.61–17.3)	23	5.66 (4.56–7.02)^3^	23	6.38 (5.25–7.74)^2^	23	16.2 (7.54–34.8)	0.004
	2		10.9 (6.79–17.6)		5.85 (4.65–7.35)^3^		6.60 (5.18–8.41)^2^		14.7 (8.00–26.9)	0.005
Average postprandial glucose response, mmol/L	1	23	8.07 (7.24–8.90)	20	7.14 (6.30–7.98)^3^	23	7.34 (6.63–8.04)^3^	21	7.41 (6.64–8.18)^3^	<0.001
	2		7.99 (7.17–8.82)		7.06 (6.23–7.90)^3^		7.34 (6.73–7.95)^3^		7.39 (6.74–8.04)^3^	<0.001
Secondary outcomes										
BMI, kg/m^2^	1	23	35.21 (32.31–38.38)	20	34.85 (31.91–38.06)^3^	20	34.22 (31.33–37.38)^3^	20	34.41 (31.44–37.65)^3^	<0.001
	2		34.33 (32.01–36.81)		33.96 (31.61–36.50)^3^		33.33 (31.12–35.70)^3^		33.50 (31.25–35.91)^3^	<0.001
Weight, kg	1	23	96.06 (86.47–106.72)	23	95.20 (85.64–105.84)^3^	23	93.44 (83.93–104.04)^3^	23	93.92 (84.22–104.73)^3^	<0.001
	2		93.84 (86.87–101.37)		92.99 (86.06–100.49)^3^		91.15 (84.58–98.23)^3^		91.58 (84.91–98.77)^3^	<0.001
Waist circumference, cm	1	22	111.48 (105.36–117.95)	23	110.00 (104.06–116.28)	23	109.07 (103.23–115.24)	23	110.23 (104.16–116.66)	0.170
	2		110.92 (104.92–17.26)		109.41 (103.72–115.42)		108.41 (103.06–114.03)		109.65 (103.72–115.93)	0.154
Fat mass, kg	1	23	42.33 (35.82–50.03)	—	—	23	40.78 (34.35–48.42)^3^	—	—	<0.001
	2		39.33 (34.39–44.97)		—		37.81 (33.08–43.21)^3^		—	<0.001
Lean mass, kg	1	23	50.11 (46.15–54.41)	—	—	23	48.90 (44.91–53.24)^3^	—	—	<0.001
	2		50.23 (47.81–52.78)		—		50.38 (47.81–53.08)		—	0.636
Visceral fat, kg	1	23	2.14 (1.62–2.84)	—	—	22	2.06 (1.57–2.70)^2^	—	—	0.025
	2		2.02 (1.67–2.44)		—		1.95 (1.64–2.33)		—	0.112
Energy, Kcal	1	20	1807 (1478–2135)	20	1534 (1230–1838)^2^	20	1576 (1299–1854)	19	1554 (1354–1754)	0.100
	2		1803 (1446–2159)		1532 (1199–1865)^2^		1576 (1233–1919)		1550 (1298–1803)	0.105
Carbohydrate, g	1	20	207 (168–246)	20	198 (163–234)	20	221 (176–265)	19	179 (154–204)	0.015
	2		211 (168–254)		202 (163–242)		225 (173–276)		183 (152–214)	0.012
Fiber, g	1	20	21 (17-26)	20	26 (21-31)^2^	20	28 (22-34)	19	22 (18-25)	0.036
	2		22 (18-27)		27 (22-32)^2^		29 (23-35)		23 (20-26)	0.050
Nonstarch polysaccharides, g	1	20	17 (13-20)	20	19 (14-22)	20	21 (16-25)	19	16 (14-19)	0.269
	2		17 (13-20)		19 (15-22)		21 (16-26)		17 (14-20)	0.285
Protein, g	1	20	77 (65–89)	20	46 (38-55)^3^	20	51 (42–60)^3^	19	73 (64–82)	<0.001
	2		77 (64–90)		46 (36-56)^3^		51 (41–62)^3^		72 (62–82)	<0.001
Fat, g	1	20	72 (55–89)	20	57 (40-73)^2^	20	52 (40-64)^2^	19	60 (50–71)	0.029
	2		70 (52–87)		55 (39-71)^2^		51 (37-64)^2^		59 (46–71)	0.031
Saturated fat, g	1	20	24 (19-32)	20	13 (9-19)^3^	20	13 (10-17)^3^	19	19 (15-24)	<0.001
	2		24 (18-32)		13 (9-17)^3^		13 (9-18)^3^		18 (14-23)	<0.001
Systolic BP, mmHg	1	23	134 (127–143)	23	132 (125–139)	23	131 (124–138)	23	130 (125–135)	0.301
	2		134 (127–142)		131 (125–137)		130 (124–136)		130 (125–135)	0.285
Diastolic BP, mmHg	1	23	81 (78–90)	23	82 (78–85)	23	82 (77–86)	23	82 (78–87)	0.667
	2		84 (78–90)		82 (78–85)		82 (77–86)		82 (78–87)	0.715
Average postprandial insulin response, mIU/L	1	23	112.04 (85.2–138)	19	92.11 (75.7–108)^2^	22	97.47 (74.7–120)	22	105.83 (83.2–129)	0.100
	2		114 (88.1–140)		94.4 (77.1–111)^2^		100 (78.0–122)		109 (86.3–131)	0.098
Total cholesterol, mmol/L	1	23	5.05 (4.49–5.61)	23	4.60 (4.17–5.02)^3^	23	4.65 (4.18–5.13)^3^	23	4.87 (4.43–5.30)	<0.001
	2		5.07 (4.58–5.56)		4.62 (4.23–5.00)^3^		4.67 (4.24–5.10)^3^		4.88 (4.47–5.29)	<0.001
HDL cholesterol, mmol/L	1	23	1.33 (1.19–1.47)	23	1.20 (1.10–1.31)^3^	23	1.21 (1.07–1.35)	23	1.24 (1.11–1.36)	0.007
	2		1.31 (1.18–1.44)		1.19 (1.08–1.30)^3^		1.19 (1.06–1.33)		1.22 (1.12–1.31)^3^	0.005
LDL cholesterol, mmol/L	1	23	2.97 (2.45–3.49)	23	2.58 (2.20–2.97)^3^	23	2.66 (2.24–3.09)^3^	23	2.87 (2.51–3.22)	<0.001
	2		3.04 (2.60–3.48)		2.65 (2.30–2.99)^3^		2.71 (2.36–3.07)^3^		2.92 (2.59–3.25)	<0.001
Total cholesterol ratio/HDL	1	23	3.93 (3.45–4.42)	23	3.92 (3.48–4.36)	23	4.01 (3.50–4.52)	23	4.02 (3.68–4.37)	0.804
	2		3.99 (3.52–4.46)		3.98 (3.58–4.39)		4.08 (3.59–4.56)		4.09 (3.78–4.40)	0.745
Triglycerides, mmol/L	1	23	1.88 (1.46–2.42)	23	1.78 (1.48–2.15)	23	1.79 (1.47–2.19)	23	1.70 (1.41–2.06)	0.140
	2		1.78 (1.41–2.25)		1.71 (1.46–2.02)		1.75 (1.45–2.10)		1.64 (1.39–1.94)	0.101
Creatinine, μmol/L	1	23	70.2 (63.2–77.3)	23	70.6 (65.1–76.0)	23	66.7 (61.5–71.8)^2^	23	68.5 (62.2–74.8)	<0.001
	2		72.9 (67.7–78.1)		73.1 (70.3–75.9)		68.9 (65.8–72.0)^2^		70.8 (66.6–75.1)	<0.001
eGFR, mL⋅min − ^2^⋅1.73 m − ^3^	1	23	97.8 (89.2–107)	23	95.4 (90.0–101)	23	103 (94.6–111)^2^	23	100 (92.1–109)	<0.001
	2		97.1 (89.8–105)		95.3 (90.3–100)		102 (96.6–109)^2^		100 (93.9–106)	<0.001
Urea, mmol/L	1	22	5.29 (4.72–5.94)	22	4.11 (3.74–4.53)^3^	22	4.26 (3.79–4.78)^3^	23	5.10 (4.69–5.56)	<0.001
	2		5.27 (4.72–5.88)		4.13 (3.75–4.43)^3^		4.29 (3.81–4.84)^3^		5.13 (4.70–5.60)	<0.001
UCR	1	22	77.5 (69.5–86.4)	22	59.3 (53.5–65.8)^3^	21	66.5 (57.01–77.48)^3^	23	77.7 (68.0–88.9)	<0.001
	2		73.9 (67.5–81.0)		57.1 (52.1–62.7)^3^		64.1 (55.4–74.1)3		74.5 (67.4–82.2)	<0.001
CRP, mg/L	1	23	3.93 (2.75–5.62)	22	3.81 (2.90–5.00)	22	3.86 (2.86–5.23)	23	4.12 (3.12–5.45)	0.782
	2		3.45 (2.47–4.80)		3.43 (2.52–4.66)		3.48 (2.51–4.82)		3.80 (2.73–5.28)	0.678

1 Outcomes included anthropometry, diet, blood pressure, and biochemistry. Data are shown as marginal means (97.5% CIs). Abbreviations: BP, blood pressure; CRP, C-reactive protein; eGFR, estimated glomerular filtration rate; TMAO, trimethylamine-N-oxide; UCR, urea to creatinine ratio.

2 *P* < 0.025 compared to baseline.

3 *P* < 0.01 compared to baseline.

### Primary outcomes

#### Glycemic control and TMAO

**FIGURE 2:**
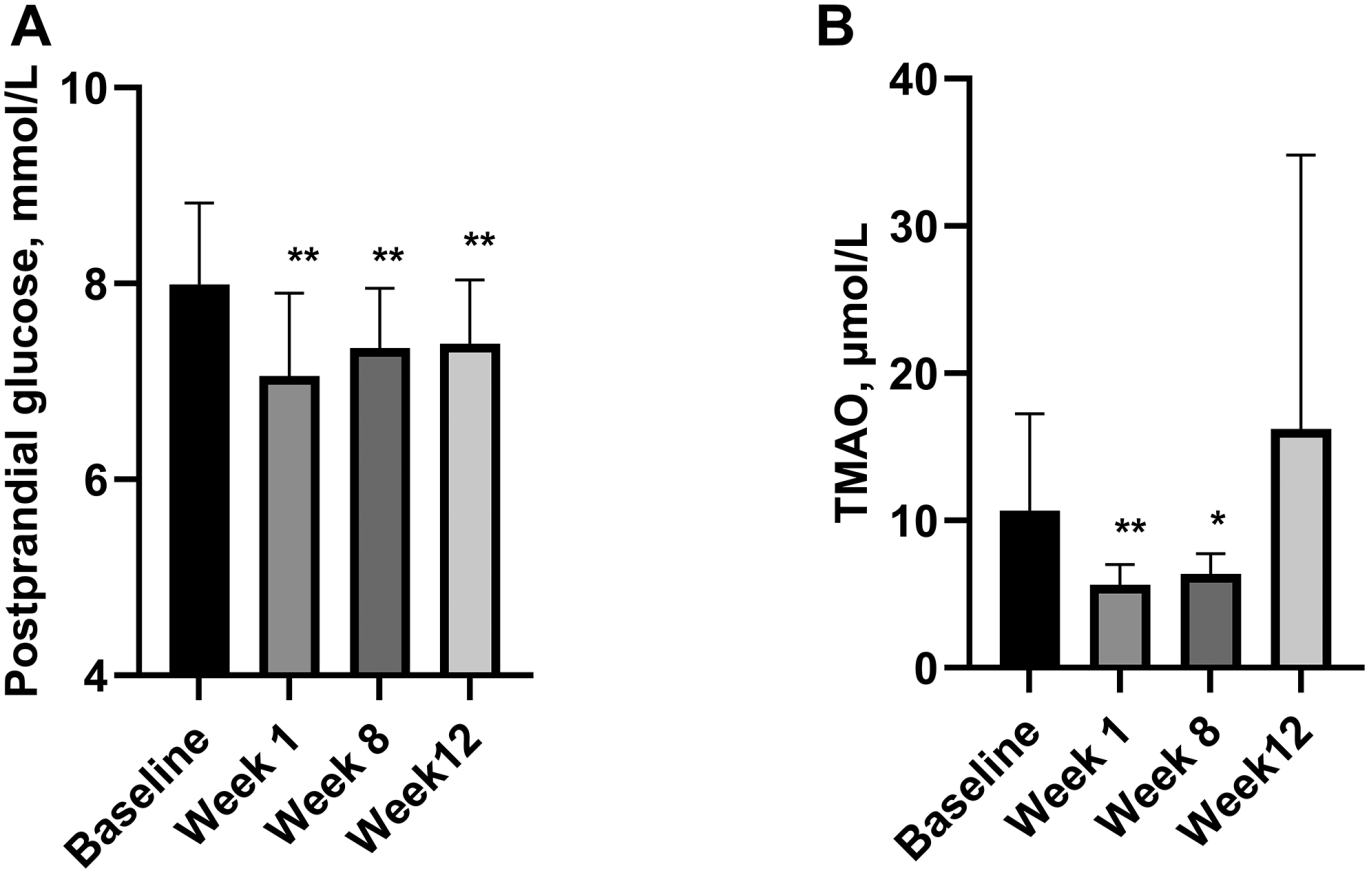
(A) Mean postprandial glucose [±SE] and (B) mean fasting TMAO [±SE] at each visit before and after an 8-week vegan diet. Data show marginal means (97.5% CIs) adjusted for age, sex, ethnicity, and weight. *Different from baseline at a *P* value < 0.025; **different from baseline at a *P* value < 0.01. Data are original to this manuscript. TMAO, trimethylamine-N-oxide.

Postprandial glucose levels were significantly (*P* < 0.025) reduced by 12% at week 1 (visit 2) and by 9% at week 8 (visit 3) compared to baseline. Levels remained significantly (*P* < 0.025) lower than baseline at week 12 (visit 4) after resumption of an unrestricted diet. Results were independent of age, sex, ethnicity, and weight (data displayed in **Figure 2** and Table 2), and were also independent of changes to diet macronutrient composition (**Supplemental Table 2**).

Plasma TMAO was significantly (*P* < 0.025) reduced by 47% at week 1 (visit 2) and by 40% at week 8 (visit 3) of the vegan diet compared to baseline. Levels rebounded and were not different from baseline at week 12 (visit 4) after resumption of an unrestricted diet. Results were independent for age, sex, ethnicity, and weight (data displayed in Figure 2 and Table 2), and were also independent of changes to diet macronutrient composition (Supplemental Table 2).

No significant correlations between changes in TMAO and changes in glucose tolerance were observed from 1 time point to the next, except for an inverse correlation between week 8 and week 12 (*r* = −0.50; *P* = 0.022). In addition, the vegan diet affected TMAO and glucose tolerance through independent pathways, as adjusting the effect of the vegan diet on glucose for TMAO or vice versa produced results that were similar in magnitude and direction to the main findings (**Supplemental Table 3**).

### Secondary outcomes

#### Bio-anthropometric measurements

Anthropometry and blood pressure measurements are presented in Table 2. There were modest but significant (*P* < 0.025) reductions in weight after 8 weeks of the vegan diet; the change in weight was mostly derived from a significant loss of fat mass (Table 2). There were no differences in waist circumference or systolic or diastolic BP in any of the models (Table 2).

Biochemistry outcomes are presented in Table 2. Total cholesterol, LDL cholesterol, and urea values were significantly (*P* < 0.025) lower at visits 2 and 3 compared to baseline in both models. HDL cholesterol was significantly (*P* < 0.025) lower at visit 2 compared to baseline in both models, but no difference was observed compared to baseline at any other visits. Urea and creatinine values, as well as the urea to creatinine ratio, were significantly (*P* < 0.025) lower and eGFR was significantly (*P* < 0.025) higher (within the normal range) after 8 weeks of a vegan diet (visit 3) compared to baseline (Table 2). No differences were observed for triglyceride and C-reactive protein measurements in any of the models.

#### Physical activity

Physical activity variables are presented in **Supplemental Table 4**. The inactive time was significantly (*P* < 0.025) lower at visit 2 but remained largely similar at visits 3 and 4 compared to baseline. The amount of light‐intensity physical activity was significantly (*P* < 0.025) higher at visit 4 compared to baseline. The amount of moderate to vigorous physical activity was similar across all time points after adjustment for wear time. Adjusting for physical activity variables did not affect the interpretation of the findings for the primary outcomes (**Supplemental Table 5**).

## Discussion

In this interventional, single-group, prospective trial, plasma TMAO levels were reduced and glucose tolerance improved after 1 week of a vegan diet in omnivores with dysglycemia or obesity, and both outcomes remained improved after 8 weeks of the intervention. The effect of a vegan diet on glucose was independent of the effect on TMAO and visa versa, suggesting the results for 1 were independent of the other. The dietary intervention also elicited a reduction in total cholesterol and improvements in markers of kidney function. To our knowledge, this is the first interventional study to investigate the impact of a vegan diet on TMAO levels in adults at risk of cardiometabolic disease.

Plant-based diets (including vegan diets) have been seen to provide nutritional advantages compared with omnivorous diets, with benefits for cardiovascular health, blood pressure and plasma lipids (24, 25). This study further demonstrates that a vegan diet substantially improves glucose tolerance whilst providing the novel finding that levels of TMAO are also reduced. These results confirm the research hypothesis, which was supported by clinical data indicating that regular consumption of meat is a risk factor for type 2 diabetes and increased levels of TMAO (5, 26). This intervention study also supports a previous, larger observational study showing that those that adhered to a vegan diet had substantially lower TMAO levels (9). Changes observed after 1 week of the vegan diet are also in line with existing evidence confirming that the gut microbiome can rapidly respond to an altered diet (27).

Whilst weight loss was not encouraged in this intervention, a mean weight loss of 2.6 kg was observed after the 8-week vegan diet. However, the effect of the vegan diet on TMAO and glucose tolerance was not affected when adjusting for weight at baseline and follow-up. Furthermore, changes in both glucose tolerance and TMAO levels were already evident at 1 week, despite greater weight loss occurring after 8 weeks. These results are in keeping with known mechanisms for the production of TMAO, which are related to the ingestion of specific nutrients, such as choline and carnitine, or to consumption of fish (9) rather than energy intake per se. The results for glucose tolerance are also supported by known mechanisms. For instance, vegan diets appear to increase the intake of protective nutrients, such as fiber (28). An increased intake of dietary fiber, predominately soluble fiber, has been shown to improve glycemic control in individuals with type 2 diabetes (29). Indeed, a number of studies have shown that nonviscous dietary fiber intake in the form of resistant starch may improve glycemic control by delaying gastric emptying, thus reducing the rate of glucose absorption and decreasing the rate of glucose uptake (26, 29). However, a previous study in those with type 2 diabetes suggested that although a vegan diet resulted in improved glycemic control over the longer-term in comparison to a standard diet (30), the benefit did not appear to be fully independent of weight loss. Therefore, the full extent to which the health benefits of a vegan diet are dependent on, or independent of, weight loss requires further research.

Importantly, this research study suggests that the effects of a vegan diet on improved glucose tolerance were independent of the reduction in TMAO, which could indicate that the reduction in TMAO precipitated by a vegan diet is not directly involved in promoting improved glucose tolerance. This is in contrast with some evidence in laboratory animal models and humans that TMAO may affect the glucose metabolism (12, 31, 32). However, it is consistent with other studies which found no link between TMAO and markers of cardiometabolic health (33). This also reflects the wider literature, where the role of TMAO as a causal marker of cardio-metabolic disease in humans is debated (34), despite the wealth of mounting animal and observational research supporting causal mechanisms or associations (35). Taken together, whilst this study suggestions a vegan diet acts to substantially reduce TMAO levels, the therapeutic importance of this reduction to improving cardiometabolic health has not been fully established and requires further research.

The effects of the vegan diet on the primary outcomes were supported by secondary outcomes, where a reduction in total cholesterol and improvements in markers of renal function were also demonstrated. An improved lipid profile has consistently been reported following a vegan diet (7). However, the finding that a vegan diet may also improve markers of renal function is novel and of potential clinical importance. Chronic kidney disease is associated with dysglycemia and is a major risk factor for chronic kidney disease (36, 30). There is emerging observational evidence that plant-based diets are associated with a reduced risk of chronic kidney disease and with better renal functioning (37), as proposed mechanisms related to plant-based diets are lower in sulfur-containing amino acids and phosphate (38). In addition, the same animal proteins that are related to the formation of TMAO, namely choline and carnitine, are also thought to increase the production of uremic toxins, which are renally eliminated and may cause impaired renal function (39). This study adds to this emerging observational evidence by suggesting that in adults at risk of cardiometabolic disease, eliminating meat from the diet results in improved renal function in the absence of chronic kidney disease. This finding therefore supports the need for further intervention research into the efficacy and effectiveness of plant-based diets in the prevention and management of chronic kidney disease.

This study has several strengths. It provides novel evidence in a high-risk cohort, which adds to the evidence around the potential efficacy of a vegan diet in type 2 diabetes and CVD prevention. Secondly, this is the first study, to our knowledge, demonstrating that a vegan diet markedly reduces levels of TMAO. Moreover, the dietary compliance rates were high throughout the intervention, further supporting the feasibility of a vegan diet intervention among this population. In fact, the acceptability of a vegan diet in clinical studies is found to be analogous to that of moderately low-fat diet (40). Nevertheless, there are some important limitations. While this study provides an initial proof of concept using an interventional before and after design, the absence of a control group means causality cannot be confirmed, and results may have been caused by co-occurring changes to other health behaviors. It is also possible that the marked reductions to saturated fat intake and increased fiber intake or the modest changes to body weight observed in this study would have provided metabolic benefits independent of a vegan diet. However, adjustment for these changes did not affect the study findings.

In conclusion, this is the first study to show that an 8-week vegan diet can elicit decreases in plasma TMAO levels when compared to baseline in individuals at risk of cardiometabolic disease, whilst also contributing to mounting experimental research showing that vegan diets lead to markedly improved glucose tolerance, independent of weight reduction. This study should be used to inform future type 2 diabetes and CVD prevention and management interventions and trials. In particular, the longer-term clinical relevance of reducing TMAO levels needs to be elucidated.

## Supplementary Material

nxab046_Supplemental_File

## Notes

This work was supported by the National Institute for Health Research (NIHR) Leicester Biomedical Research Centre, Leicester, UK (to SA, MJD, GJHB, DB, TS, NPD, AVR, ACS, and TY); NIHR Applied Research Collaboration East Midlands (to KK).

Author disclosures: The authors report no conflicts of interest.

The funding source had no role in study design, data collection, data analysis, data interpretation or writing of the report. The views expressed are those of the authors and not necessarily those of the National Health Service, the NIHR or the Department of Health and Social Care.

Supplemental Tables 1–5 are available from the “Supplementary data” link in the online posting of the article and from the same link in the online table of contents at https://academic.oup.com/jn/.

Abbreviations used: CVD, cardiovascular disease; eGFR, estimated glomerular filtration rate; GEE, generalized estimating equations; HbA1c, glycated hemoglobin; OGTT, oral-glucose-tolerance test; tAUC, total AUC; TMA, trimethylamine; TMAO, trimethylamine-N-oxide.
