# Supplementary material for: Evaluation of an 8-Week Vegan Diet on Plasma Trimethylamine-N-Oxide and Postchallenge Glucose in Adults with Dysglycemia or Obesity
Source: J Nutr. 2021 Mar 30;151(7):1844–53. doi: 10.1093/jn/nxab046 (PMC8245890; doi:10.1093/jn/nxab046)
Supplement: nxab046_Supplemental_File [file nxab046_supplemental_file.docx]

**Online Supplementary Material**

Evaluation of an 8-week vegan diet on plasma Trimethylamine-N-Oxide and post-challenge glucose in adults with dysglycemia or obesity.

Stavroula Argyridou ^1, 2^, Melanie J Davies ^1, 2^, Gregory JH Biddle ^1,2^, Dennis Bernieh ^3^, Toru Suzuki ^3^, Nathan P Dawkins ^1,2^, Alex V Rowlands ^1,2^, Kamlesh Khunti ^1, 4^, Alice C Smith ^5^, Thomas Yates ^1, 2*^

^1^Diabetes Research Centre, University of Leicester, Leicester General Hospital, Gwendolen Rd, Leicester LE5 4PW, UK

^2^NIHR Leicester Biomedical Research Centre, University of Leicester, Leicester LE1 7RH, UK

^3^Department of Cardiovascular Sciences, University of Leicester, Leicester, NIHR Leicester Biomedical Research Centre, Leicester, UK

^4^NIHR Collaboration for Leadership in Applied Health Research and Care—East Midlands, University as Leicester, Leicester, LE1 7RH, UK

^5^Leicester Kidney Lifestyle Team, Department of Health Sciences, University of Leicester, LE1 7RH UK

Table of Contents

[Supplemental Table 1: Compliance 2](#_Toc61508874)

[Supplemental Table 2: The effects of diet composition on TMAO (μmol/L) and glucose tAUC (mmol/L); mean (97.5% CI)) 2](#_Toc61508875)

[Supplemental Table 3: Sensitivity analysis (mean (97.5% CI)) 3](#_Toc61508876)

[Supplemental Table 4: The effect of the intervention on secondary outcomes (physical activity; mean (97.5% CI)) 4](#_Toc61508877)

[Supplemental Table 5: The effects of physical activity on TMAO (μmol/L) and glucose tAUC (mmol/L); mean (97.5% CI)) 4](#_Toc61508878)

# Supplemental Table 1: Compliance

| **Point of contact** | **Week 1** | **Visit 2** | **Week 2** | **Week 3** | **Week 4** | **Week 5** | **Week 6** | **Week 7** | **Week 8** |
| --- | --- | --- | --- | --- | --- | --- | --- | --- | --- |
| **Participants Responded** | 13 | 21 | 11 | 16 | 15 | 12 | 11 | 15 | 22 |
| **Mean score (out of 10)** | 9.92 ±  0.28 | 9.90 ± 0.30 | 9.91 ± 0.30 | 9.94 ± 0.25 | 9.93 ± 0.26 | 9.75 ± 0.62 | 9.82 ± 0.60 | 10 ± 0.00 | 9.45 ± 1.18 |

*Participants were contacted via telephone calls by the study RD between the visits.

# Supplemental Table 2: The effects of diet composition on TMAO (μmol/L) and glucose tAUC (mmol/L) after adjustment for macronutrient intake; mean (97.5% CI))

| **Variables** | **Model** | **n** | **Baseline** | **n** | **Week 1** | **n** | **Week 8** | **n** | **Week 12** | **P for model** |
| --- | --- | --- | --- | --- | --- | --- | --- | --- | --- | --- |
| TMAO, μmol/L | 3 | 23 | 9.38 (5.11-17.2) | 23 | 5.22 (3.76-7.23) | 23 | 6.79 (4.59-10.05) | 23 | 16.9 (9.28-30.82) | **<0.001** |
| Glucose tAUC, mmol/L | 3 | 23 | 8.10 (7.26-8.95) | 20 | 6.94 (5.92-7.96) | 23 | 7.17 (6.47-7.86) | 21 | 7.40 (6.64-8.16) | **<0.001** |

* P <0.025 compared to baseline, ** P <0.01 compared to baseline

Model 3: Model 2 + macronutrient intake (carbohydrates, protein, fat)

# Supplemental Table 3: Sensitivity analysis (mean (97.5% CI))

| **Variables** | **Model** | **n** | **Baseline** | **n** | **Week 1** | **n** | **Week 8** | **n** | **Week 12** | **P for model** |
| --- | --- | --- | --- | --- | --- | --- | --- | --- | --- | --- |
| TMAO, μmol/L | 1 | 23 | 10.6 (6.51-17.1) | 23 | 5.42 (4.30-6.8)** | 23 | 6.39 (5.26-7.75) | 23 | 17.5 (7.98-38.4) | **0.003** |
|  | 2 |  | 10.9 (6.66-17.9) |  | 5.40 (4.08-7.15)** |  | 6.66 (5.17-8.58) |  | 15.7 (8.5-29.0) | **0.004** |
| Glucose tAUC, mmol/L | 1 | 21 | 8.07 (7.25-8.90) | 20 | 7.10 (6.25-7.95)** | 23 | 7.31 (6.61-8.02)** | 21 | 7.46 (6.69-8.22)** | **<0.001** |
|  | 2 |  | 8.00 (7.18-8.83) |  | 7.03 (6.19-7.88)** |  | 7.32 (6.71-7.93)** |  | 7.43 (6.79-8.07)** | **<0.001** |

* P <0.025 compared to baseline, ** P <0.01 compared to baseline; n-number of participants with valid data per visit

Model 1: adjusted for TMAO when assessing glucose tAUC and vice versa

Model 2: age, sex, ethnicity, weight, TMAO when assessing glucose tAUC and vice versa

# Supplemental Table 4: The effect of the intervention on secondary outcomes (physical activity; mean (97.5% CI))

| **Variables** | **Model** | **n** | **Baseline** | **n** | **Week 1** | **n** | **Week 8** | **n** | **Week 12** | **P for model** |
| --- | --- | --- | --- | --- | --- | --- | --- | --- | --- | --- |
| Wear time, min/day | 1 | 21 | 1365  (1302-1431) | 21 | 1367  (1307-1430) | 23 | 1384  (1330-1440) | 22 | 1366  (1309-1424) | 0.082 |
|  | 2 |  | - |  | - |  | - |  | - | - |
| Inactive time, min/day | 1 | 21 | 765  (701-830) | 21 | 733  (661-805)* | 23 | 759  (706-812) | 22 | 766  (715-817) | 0.132 |
|  | 2 |  | 755  (721-790) |  | 725  (680-771)* |  | 765  (731-798) |  | 756  (732-781) | **0.026** |
| Light‐intensity physical activity, min/day | 1 | 21 | 168  (150-190) | 21 | 177  (157-201) | 23 | 175  (156-196) | 22 | 188  (168-210)** | **<0.001** |
|  | 2 |  | 168  (151-187) |  | 177  (158-199) |  | 176  (158-196) |  | 187  (170-207)** | **<0.001** |
| MVPA, min/day *** | 1 | 21 | 28  (19-40) | 21 | 23  (15-36)* | 23 | 24  (18-33) | 22 | 25  (18-34) | 0.063 |
|  | 2 |  | 27  (19-37) |  | 22  (16-29) |  | 22  (17-29) |  | 23  (18-29) | 0.142 |

* P <0.025 compared to baseline; ** P <0.01 compared to baseline; ***MVPA-Total MVPA of at least 1min bouts; Model 1: unadjusted; Model 2: age, sex, ethnicity, weight + wear time; - = Not applicable

# Supplemental Table 5: The effects of physical activity on TMAO (μmol/L) and glucose tAUC (mmol/L); mean (97.5% CI))

| **Variables** | **Model** | **n** | **Baseline** | **n** | **Week 1** | **n** | **Week 8** | **n** | **Week 12** | **P for model** |
| --- | --- | --- | --- | --- | --- | --- | --- | --- | --- | --- |
| TMAO, μmol/L | 3 | 23 | 8.87  (6.12-12.9) | 23 | 5.64  (4.40-7.22) | 23 | 6.73  (5.14-8.80) | 23 | 14.4  (8.11-25.5) | **0.014** |
| Glucose tAUC, mmol/L | 3 | 23 | 7.95  (7.05-8.85) | 20 | 7.05  (6.18-7.92)** | 23 | 7.33  (6.65-8.00)** | 21 | 7.43  (6.72-8.13)* | **<0.001** |

* P <0.025 compared to baseline, ** P <0.01 compared to baseline

Model 3: Model 2 + physical activity (sedentary time, MVPA and wear time)
